# Measurements of quality of village-level care and patients’ healthcare-seeking behaviors in rural China

**DOI:** 10.1186/s12889-021-11946-8

**Published:** 2021-10-17

**Authors:** Sihang Rao, Hao Xue, Dirk E. Teuwen, Haonan Shi, Hongmei Yi

**Affiliations:** 1 China Center for Agricultural Policy, School of Advanced Agricultural Sciences, Peking University, Room 408B, Wangkezhen Building, No. 5, Yiheyuan Road, Haidian, Beijing, 100871 China; 2 Stanford Center on China’s Economy and Institutions, Stanford University, California, USA; 3 Medical Sustainability, UCB, Brussels, Belgium; 4 Department of Neurology, Ghent University Hospital, Ghent, Belgium; 5 Business Development Center, Red Cross Society of China, Beijing, China

**Keywords:** Primary health care, Healthcare-seeking behaviors, Credence goods, Quality indicators, Rural China

## Abstract

**Background:**

Although the progress in global health initiatives has improved the availability of primary health care (PHC), unqualified healthcare remains a serious challenge in low- and middle-income countries, where PHC is often underutilized. This study examines factors associated with patients’ healthcare-seeking behaviors in rural Chin—seeking healthcare at village-level PHC providers, at higher-level health facilities, self-medicating, and refraining from seeking medical help. We focus on provider-side factors, including (1) the unobservable quality indicator, (2) the observable quality indicator, and (3) the observable signal indicator.

**Methods:**

We analyzed 1578 episodes of healthcare-seeking behaviors of patients with diarrhea or cough/runny nose symptom from surveys conducted in July 2017 and January 2018 in 114 villages of the Yunnan province. We investigated the correlation between quality-related factors with patients’ healthcare-seeking behaviors by multinomial logit regression.

**Results:**

We found that rural patients were insensitive to the unobservable quality of healthcare providers, as measured by standardized clinical vignettes, which might be attributable to the credence nature of PHC. The observable quality indicator, whether the clinician has received full-time junior college formal medical education, was associated with patients’ healthcare choices. Patients, however, were more likely to select healthcare based on the observable signal indicator, which was measured by the availability of medicines. Additionally, the observable signal indicator had no significant association with two quality indicators. Notably, socioeconomically-disadvantaged patients relied more on the village-level PHC, which emphasized the role of PHC in promoting the welfare of rural populations.

**Conclusions:**

Our study found an inconsistency between objective quality of healthcare provided by providers and subjective quality perceived by patients. Patients could not identify the actual quality of PHC precisely, while they were more likely to make decisions based on the observable signal indicator. Therefore, the quality of PHC should be more observable to patients. This study not only supplements the literature on healthcare-seeking choices by examining four types of behaviors simultaneously but also clarifies rural patients’ perceptions of the quality of PHC for policy decision-making on increasing the utilization of PHC and improving the medical welfare of the vulnerable.

**Supplementary Information:**

The online version contains supplementary material available at 10.1186/s12889-021-11946-8.

## Background

### Introduction

Primary healthcare (PHC) is a patient’s first point of contact with the healthcare system and provides comprehensive local-based clinical care and public health services. PHC is regarded as the most effective and efficient whole-of-society approach to promoting physical and mental health and welfare, at both the individual and population levels [1, 2]. Hence, growing numbers of global health promotion programs have been initiated in response to the Alma-Ata Declaration of 1978 [3]. Many countries have been committing resources to establish and strengthen their PHC systems [3].

However, many people do not seek clinical care from PHC facilities when they are supposed to do. First, it is common for patients to bypass nearby PHC for providers that are farther away, not only in low- and middle-income countries (LMICs) [4, 5], but even in high-income countries where competent PHC are accessible [6, 7]. These health-seeking behaviors incur a loss of earnings, additional economic costs, and a waste of resources. Second, many patients, particularly in LMICs, choose to self-medicate. Improper self-medication, mainly via the inappropriate use of prescription-only medicines (POMs) (e.g.*,* antibiotics), causes potential health hazards. A systematic review of 34 studies reported a 38.8% overall prevalence of antimicrobial self-medication in LMICs is 38.8% with 34.1% in the Middle East, 38.0% in Asia, 40.6% in Sub-Saharan Africa, and 44.1% in South America [8]. This phenomenon also exists in high-income economies, however, where the prevalence is relatively lower due to strict regulations on drug sales—7% in European Union [9] and 5% in the United States [10].

Concern regarding the PHC quality in LMICs is a major factor to motivate patients to seek care elsewhere [11]. Although progress in global health initiatives has improved the availability of PHC, low-quality PHC remains a serious issue in LMICs [11]. More importantly, it is not clear which quality-related indicators matter to patients. Indeed, the provision of healthcare, as a typical credence good, is influenced by the information asymmetry between the provider and patient. Credence goods are defined as goods and services of which the provider has more knowledge than the patients concerning knowledge of the quality of the care offered, than the patients [12]. Therefore, patients are unable to determine the actual quality of care prescribed or provided.

Previous studies on individual healthcare-seeking behaviors have mainly concentrated on two types of quality proxies. The first type includes the availability of medicine and medical equipment [4, 5]. However, this quality proxy has been criticized due to over-treatment and out-of-stock situations [13]. This proxy functions as the signal that the provider sends to the patient regarding quality; however, this proxy is not necessarily indicative of high quality. The other type of proxy, which is also observable for patients, is the clinician’s professional literacy level (e.g., their education, qualification, and training levels) [14]. However, this proxy does not consider the skills acquired in practice or the potential behavioral distortions of the provider due to inappropriate incentives.

Methods that more accurately measure providers’ quality of care have only recently been developed. These methods, which include vignettes, clinical observations, chart abstraction, and standardized patients, have been adopted and validated in different studies in LMICs [11, 15, 16]. Precisely, the unobservable quality of care is usually measured by the adherence to clinical checklists, accurate diagnoses, and appropriate managements. Despite their advantages, these methods have rarely been applied in studies on healthcare-seeking behaviors.

This study focused on village clinics (VCs), which are at the front line of the PHC system in rural China. Since the initiation of health reforms in 2009, China has implemented several initiatives to improve the quality of village-level PHC and encourage rural residents to seek local healthcare first. Nevertheless, village-level PHC remains underutilized [17, 18]. Therefore, this study has two primary objectives. First, we attempt to describe the healthcare-seeking behaviors of patients, including seeking services at VCs or higher-level medical facilities (or bypassing), self-medicating, and refraining from medical help (or self-healing). Second, we examine and identify factors associated with patients’ healthcare-seeking behaviors, in terms of the unobservable and observable quality indicators, and the observable signal indicator of healthcare.

Using village clinicians’ competence in the process of diagnosis and treatment, as measured by vignettes, we concluded that patients were insensitive to the more accurate but unobservable quality indicator. The observable quality indicator, as measured by whether the clinician has received a full-time formal junior college medical education, was related to the patient’s choice. Moreover, the availability of medicines was highly valued by patients. While the observable signal seemingly functions as a quality indicator for patients, it does not represent the actual quality of healthcare. We also found that well-educated patients were more sensitive to the unobservable quality indicator than less-educated patients. These findings supported the argument that the healthcare market is part of the credence goods market.

This study contributed to the literature on PHC and the determinants of healthcare-seeking behaviors in the following aspects. First, we focused on village-level PHC, which is the most vulnerable part of the rural health network [19] and has received scant attention thus far. Second, we analyzed patients’ healthcare-seeking behaviors under a credence nature of healthcare framework. This view has rarely been discussed, and the findings could facilitate investment-related decision-making to improve the utilization of PHC. Third, we considered four types of healthcare-seeking behaviors to provide ample evidence, while most studies were limited to one or two behavior types.

### Three-level rural healthcare system and utilization of village-level PHC in rural China

**Fig. 1:**
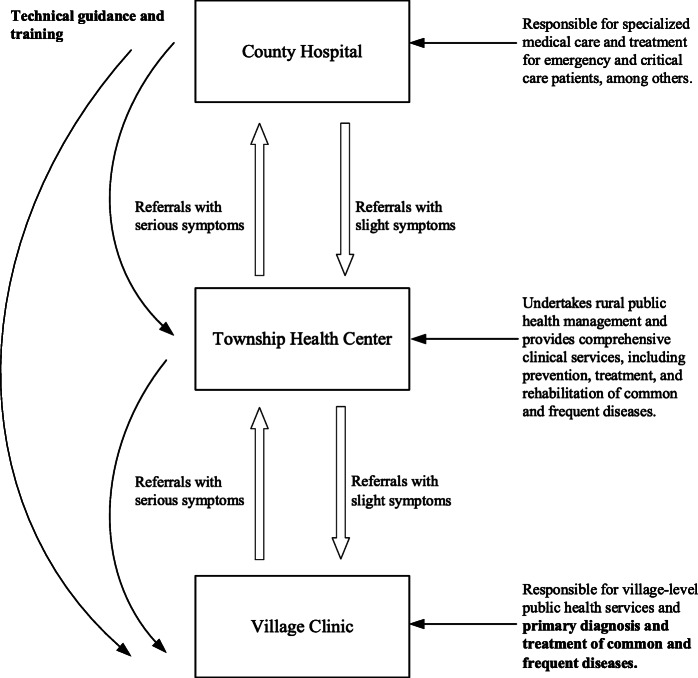
Three-level healthcare system in rural China

In China, the urban healthcare system and rural healthcare system function parallelly. Although Chinese patients are free to choose healthcare providers without adhering to compulsory gatekeeping regulations [20], only around 2.7% of rural patients bypassed the rural healthcare system to visit a municipal or provincial hospital [21]. The rural Chinese healthcare system is comprised of three levels—county hospitals (CHs), township health centers (THCs), and VCs. Since the initiation in 2009, the health reform has underlined the need for the division and collaboration between these medical facilities [22]. CHs, which form the system’s top level, undertake expensive and specialized services for county residents, especially those with serious and emergent symptoms. THCs and VCs are PHC providers. THCs, which form the system’s middle level, play a pivotal role in offering comprehensive clinical care of common and frequent diseases and providing technical guidance and supervision for village clinicians. VCs, which form the system’s bottom-level, fall under the management and supervision of THCs and facilitate the provision of primary care for common communicable and non-communicable diseases (e.g., diarrhea and cold) [23]. In addition to clinical services, THCs and VCs are also responsible for the provision of rural public health services. Figure 1 provides a comprehensive description of the three-level healthcare system in rural China.

To enhance the rural healthcare system’s efficiency, the Chinese government has emphasized village-level PHC since 2009. In addition to financial investment, infrastructure building, and clinician training [17, 24], the government has attempted to rebuild the referral system and encourage patients to use village-level PHC. In 2015, the government issued guidelines on promoting the hierarchical diagnosis and treatment system [25]. The government expects rural patients to fist seek care at lower-level facilities and only recommends higher-level hospital intervention for patients with severe symptoms that cannot be managed by PHC providers (see Fig. 1).

However, this referral policy is not mandatory and does not have a distinct effect [17]. In the absence of compulsory gatekeeping, many rural patients bypass the first-contact care at their local PHC to seek better healthcare at the higher-level facilities [26]. After peaking in 2013, the total number of visits to VCs has experienced a downward trend [27]. Specifically, it decreased by 16.9% between 2013 and 2018, whereas the number of visits to THCs and CHs increased by 10.8 and 29.9%, respectively, in the corresponding period. Meanwhile, many rural patients choose to self-medicate due to the acquisition of POMs without prescriptions and overprescribing behaviors of clinicians [28]. The proportion of rural patients aged 45 years and over who self-medicated over one month increased from 45.5% in 2011 to 54.8% in 2015 [29]. These behaviors lower the utilization rate of VCs and undermine the whole health system. Therefore, it is crucial to analyze the determinants of individual healthcare-seeking behaviors and provide empirical evidence for policies that aim to promote the village-level PHC.

Although China’s village-level PHC has greatly contributed to improving the rural population’s health using scarce resources in the past [30], the low-quality of village-level PHC has continued for a long time in rural China [18]. The low quality of PHC could be attributed to village clinicians’ limited education and qualification levels [17, 19]. Some studies have also used standardized patients or vignettes to find empirical evidence on the low-quality of PHC, reflected as low adherences to clinical checklists, low rates of correct diagnosis and treatment, and high rates of inappropriate antibiotic prescription [15, 31–33].

Although many studies have consistently concluded that the poor-quality PHC drives rural patients to either opt for care at a higher-level facility or self-medicate in China, these studies have rarely measured quality accurately. For example, empirical studies have confirmed that patients’ perceptions of quality and observable signals (e.g., medicine and medical equipment availability) are related to patients’ healthcare-seeking behaviors [26]. However, the previous literature has shown no significant correlation between patients’ self-reported assessment of PHC quality and record-based quality [34] or clinicians’ medical knowledge [35]. Studies have also found no association between observable signals and healthcare quality [36]. This potential inconsistency between the observable indicators and unobservable quality might mislead the policy reforms needed to improve rural PHC.

## Methods

### Sampling

Data were taken from a concurrent randomized controlled trial to evaluate the impact of training on village clinicians’ knowledge and performance in rural Yunnan, a South-western province in China [37]. In 2017, the per capita GDP of Yunnan province was USD 5068, which was 42 percentage points lower than that of the national level (USD 8777) [38]. The rural population of the three sampled prefectures—Dali, Yuji, and Quinn—was 6.5 million, 20.0% of Yunnan’s rural population [39].

We selected village clinicians in the first round of the survey in July 2017 by three steps. In the first step, we selected 10 counties from the 30 counties in the three prefectures, excluding 3 urban counties and 11 counties that had more than a 20.0% non-Han population. Thus 16 counties (3, 5, and 8 counties from Dali, Yuki, and Qujing) were eligible for sampling. Subsequently, we randomly selected 10 counties (2, 3, and 5 counties, respectively) from the 16 counties. In the second step, we selected VCs. First, we excluded urban communities and their clinics in each county. Subsequently, the sample size of the VCs in each county was determined by the proportion of the VCs in all selected counties. Finally, we randomly selected 330 VCs from 10 counties. In the third step, we selected village clinicians. First, we excluded clinicians who only prescribed traditional Chinese medicine or did not provide medical care. Second, we selected the leading clinician who was the main provider of medical care at each clinic. If there were two or more leading clinicians, one was randomly selected from the list. Accordingly, a total of 330 village clinicians were selected.

A follow-up survey was conducted in January 2018, where approximately one-third (114) of the baseline villages were randomly selected to conduct the household survey. First, we collected the household roster from the VC of each village. Second, we excluded households that were more than 3 km from the nearest road that was accessible by car and those that had no available adult member on the survey day. Then, we divided the eligible households into two groups—households with a chronic patient (hypertension or/and diabetes) and households without chronic patients. Subsequently, we randomly selected six households from the households with chronic patients.^1^ If there were less than six eligible households, the rest were selected from the other group. We collected information on all members of each selected household, which yielded a sample of 692 households in 114 villages.^2^ A total of 235 households (34.0%) had at least one chronic patient. The prevalence of hypertension and diabetes in adults in the sampled adults was 14.0 and 2.6%, respectively, which was similar to the prevalence in western rural China of 14.9 and 2.8%, respectively, in 2018 [21].

**Fig. 2:**
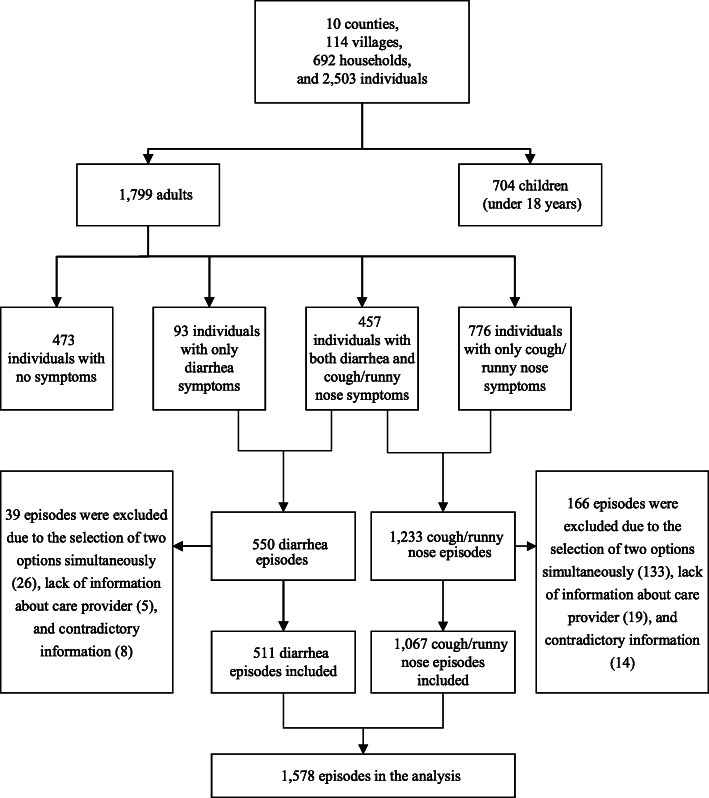
Sample selection process

Figure 2 illustrates the sample selection process. The selected households contained 2503 individuals: 1799 (71.9%) adults and 704 (28.1%) children. We excluded children from the sample because we could not identify which primary caregivers played the role of decision-maker when seeking healthcare for the children. Of the 1799 adults, 473 (26.3%) adults have no symptoms of diarrhea or a cough/ runny nose in the previous year since the survey, while 93 (5.2%) adults had only diarrhea 776 (43.1%) had only a cough/runny nose, and 457 (25.4%)adults, had both diarrhea and a cough/ runny nose; thus, 1783 episodes were obtained. We further excluded 205 episodes with unidentified information regarding patients’ healthcare-seeking choices and the medical facility that they had visited, thus gaining a total sample of 1578 episodes for analyses. Table A1 in the Additional file 1 shows the comparative descriptive statistics between the included and excluded sample.

### Data collection

The data collection on the providers concerned the village clinician level and clinic level. In the clinician-level survey, we collected the village clinicians’ demographic and socioeconomic information. We also used standardized clinical vignettes to measure the competence of village clinicians. A vignette is a hypothetical case in which a well-trained interviewer plays an unblinded patient with a specific disease and provides a brief description of their symptoms. In this simulated scenario, the clinician needs to diagnose, prescribe, and treat this “patient” as they would under normal circumstances. During the process of diagnosis and treatment, the interviewer responds with predetermined standard answers to the questions posed by the clinician [11] The competence of the village clinician was evaluated based on three aspects of clinical practice: diagnostic process quality, diagnosis accuracy, and case management. The assessment standards conformed to international and Chinese National Practice Guidelines [16, 32]. This method allowed us to capture the clinicians’ competence in actual cases more accurately than merely assessing the clinicians’ education, qualifications, and training level [11]. We presented four vignettes to each clinician—diarrhea, cold, hypertension, and asthma. We determined correct/partially correct medication for the corresponding vignette (diarrhea or cold) to measure the accurate but unobservable quality.

The clinic-level survey collected basic information of the VCs, including drug stock, the number of patients served and referred to by the sampled clinic in the previous month since the survey, the number of permanent residents within 5 km of the VC, and the distance and the availability of shuttles from the VC to the THC.

For the household-level survey, the respondent was the head of the household. If they were absent, we collected responses from another family member who was the most familiar with their family’s activities. We first collected the information on the household, including whether the family was related to the village clinician, whether the household was officially identified as an impoverished household, and the list of medicines stored at home. Second, we collected the information on the demographic and socioeconomic information of each family member. Then, we asked a series of questions about the healthcare-seeking experiences of each family member in the previous year. In addition to hypertension and diabetes, we focused on the symptoms of diarrhea and a cough/ runny nose, which were commonly diagnosed and treated in VCs. For each symptom, we first asked the head of household to report the number of family members with symptoms. Subsequently, we collected detailed information about each family member’s healthcare-seeking behavior during their most recent sickness episode in which they had such symptoms. Specifically, we asked about the severity of the sickness, healthcare-seeking choices. The members who first sought formal healthcare were further asked about the healthcare facilities that they selected.

### Empirical methods

We use a multinomial logit regression to analyze the determinants of different healthcare-seeking behaviors from patients’ and providers’ perspectives. The model is specified as:

$$ \mathit{\log}\frac{\mathit{\Pr}\left({y}_{idjc}=z\right)}{\mathit{\Pr}\left({y}_{idjc}= 1\right)}={\beta}_0+{\beta}_1\;{Qu}_{djc}+{\beta}_2\;{Qo}_{jc}+{\beta}_3\;{Qs}_{jc}+{\gamma}_1\;{\boldsymbol{VC}}_{jc}+{\gamma}_2\;{\boldsymbol{P}}_{idjc}+{\alpha}_d+{\alpha}_c+{\varepsilon}_{idjc},z= 2, 3, 4. $$

where *y*_*idjc*_ is a categorical variable denoting the healthcare-seeking behavior of the patient *i* with symptom *d* from village *j*, county *c*. This outcome variable takes values from 1 to 4, representing first seeking formal healthcare from VCs (baseline group), first seeking formal healthcare from higher-level facilities, including THCs, CHs, and hospitals in urban areas (bypassing group), taking medicines only (self-medicating group), and refraining from seeking medical help (self-healing group), respectively. This study focuses on two common symptoms, diarrhea and a cough/runny nose, which should be diagnosed and treated in VCs in line with the guidelines on the rural hierarchical diagnosis and treatment system. Thus, although THCs are PHC providers in principle, we still define visiting THCs first as a bypassing event.

*Qu*_*djc*_*, Qo*_*jc*_*,* and *Qs*_*jc*_ are the three quality-related indicators in the main analysis. *Qu*_*djc*_ denotes whether the clinician gives correct/partially correct medication in the corresponding vignette (diarrhea or cold), reflecting the true but unobserved quality of the provider. *Qo*_*jc*_ denotes whether the clinician has received full-time formal junior college medical education and captures the observable quality of the provider. *Qs*_*jc*_ is the observable signal indicator—the variety of Western medicine in stock. To mitigate endogeneity, we also control a vector of the providers’ characteristics, ***VC***_***jc***_, which includes the geographic distance and the availability of a shuttle from the clinic at village *j* to the THC, the number of village clinicians per 1000 people, the referral rate of patients, and the number of visits to the VC.

***P***_***idjc***_ includes the individual and family characteristics of patient *i*. We use individual-level socioeconomic variables, including age, gender, education, non-farm employment, the presence of hypertension or diabetes, and whether the patient is a village leader. The family-level characteristics include whether the household head is a relative of village clinicians, and whether the family is officially identified as an impoverished household. *α*_*d*_ and *α*_*c*_ are symptom and county fixed effects, respectively.

## Results

### Healthcare-seeking behaviors

Table 1 presents patients’ healthcare-seeking behaviors from the 1578 episodes, including 511 diarrhea episodes and 1067 cough/ runny nose episodes. Overall, 59.5% of the episodes contained patients who self-medicate. There were 23.5, 8.1, and 8.9% of episodes that contained patients who sought medical care at VCs, bypass VCs, and self-heal, respectively. The pattern of healthcare-seeking behaviors was different between the two symptoms. Specifically, the likelihood of patients with diarrhea seeking medical care at VCs or bypassing VCs was lower than that of patients with a cough/ runny nose. Meanwhile, the likelihood of patients with diarrhea self-medicating was higher than that of patients with a cough/ runny nose.

**Table 1:** Healthcare-seeking behaviors: *n (%)*

Outcomes	Total (1)	Diarrhea (2)	Cough/runny nose (3)	Difference ^a^ (4) = (2)–(3)
**Number of episodes**	1578	511	1067	
Seeking healthcare at VCs	371 (23.5)	75 (14.7)	296 (27.7)	−13.1***
Bypassing	128 (8.1)	30 (5.9)	98 (9.2)	−3.3**
Self-medicating	939 (59.5)	352 (68.9)	587 (55.0)	13.9***
Self-healing	140 (8.9)	54 (10.6)	86 (8.1)	2.5

*Note:* ^a^ Column (4) presents the percentage point difference in four healthcare-seeking behaviors between diarrhea and cough/ runny nose episodes. Significance level: *** *p* < 0.01, ** *p* < 0.05

### Characteristics of patients and providers

Table 2 presents the descriptive statistics of the characteristics of patients. Overall, 27.7, 49.7, 43.5, 7.3, and 13.2% of the selected patients were over 60 years of age, male, non-farm jobholders, village leaders, and relatives of village clinicians, respectively. These characteristics showed no significant difference across the four groups.

**Table 2:** Descriptive statistics of *patients’* characteristics: *mean (SD)*

	Total (1)	Seeking healthcare at VCs (2)	Bypassing (3)	Self-medicating (4)	Self-healing (5)	Difference in Mean		
						(6) = (2)–(3)	(7) = (2)–(4)	(8) = (2)–(5)
***Patients’ s characteristics***								
Elderly (over 60 = 1)	0.277 (0.448)	0.294 (0.456)	0.336 (0.474)	0.261 (0.439)	0.286 (0.453)	−0.042	0.033	0.008
Male (yes = 1)	0.497 (0.500)	0.499 (0.501)	0.461 (0.500)	0.493 (0.500)	0.557 (0.499)	0.038	0.006	−0.058
Education								
Illiterate	0.302 (0.459)	0.310 (0.463)	0.398 (0.492)	0.297 (0.457)	0.221 (0.417)	−0.088*	0.013	0.089**
Primary school	0.369 (0.483)	0.391 (0.489)	0.352 (0.479)	0.363 (0.481)	0.364 (0.483)	0.039	0.028	0.027
Junior middle school	0.256 (0.437)	0.253 (0.436)	0.188 (0.392)	0.262 (0.440)	0.286 (0.453)	0.066	−0.009	−0.032
Senior middle school	0.048 (0.213)	0.038 (0.191)	0.055 (0.228)	0.047 (0.211)	0.071 (0.258)	−0.017	−0.009	− 0.034
College or higher education	0.026 (0.159)	0.008 (0.090)	0.008 (0.088)	0.031 (0.173)	0.057 (0.233)	0.000	−0.023**	−0.049***
Hypertension or diabetes (yes = 1)	0.163 (0.369)	0.224 (0.417)	0.172 (0.379)	0.132 (0.339)	0.200 (0.401)	0.052	0.092***	0.024
Non-farm jobholder (yes = 1)	0.435 (0.596)	0.412 (0.493)	0.484 (0.502)	0.431 (0.496)	0.479 (0.501)	−0.072	−0.019	−0.066
Village leader (yes = 1)	0.073 (0.260)	0.086 (0.281)	0.063 (0.243)	0.066 (0.248)	0.093 (0.291)	0.024	0.020	−0.007
Self-reported severity of a symptom								
Mild	0.511 (0.500)	0.340 (0.474)	0.203 (0.404)	0.575 (0.495)	0.814 (0.390)	0.136***	−0.235***	− 0.475***
Moderate	0.234 (0.423)	0.251 (0.434)	0.148 (0.357)	0.260 (0.439)	0.093 (0.291)	0.102**	−0.009	0.158***
Severe	0.255 (0.436)	0.410 (0.492)	0.648 (0.479)	0.165 (0.371)	0.093 (0.291)	−0.239***	0.245***	0.317***
Relative of village clinicians (yes = 1)	0.132 (0.338)	0.113 (0.317)	0.109 (0.313)	0.147 (0.354)	0.100 (0.301)	0.004	−0.034	0.013
Impoverished household (yes = 1)	0.313 (0.464)	0.375 (0.485)	0.289 (0.455)	0.291 (0.454)	0.321 (0.469)	0.086*	0.084***	0.053

Note: Significance level: *** *p* < 0.01, ** *p* < 0.05, * *p* < 0.1

The average education level of our sample was relatively low. Over 90% of patients had not completed senior middle school. The highest education level of the patients seeking care at professional medical facilities was lower than the level of others. There were 51.1, 23.4, and 25.5% of patients with mild, moderate, and severe symptoms, respectively. Patients in the baseline and bypassing group were more likely to report a severe symptom than those in the other two groups. Moreover, 31.3% of the patients belonged to impoverished households, and this proportion was the highest in the baseline group.

Table 3 presents the descriptive statistics of the characteristics of the PHC providers located in the village of the patients. The rate of providers who issued correct/partially correct medication in the corresponding vignettes was 0.501 on average and 6.3% of clinicians had received full-time formal junior college medical education. The variety of Western medicines in stock was 78.976 in the whole sample, and the variety in the baseline group far exceeded the other three groups.

**Table 3:** Descriptive statistics of *providers’* characteristics: *mean (SD)*

	Total (1)	Seeking healthcare at VCs (2)	Bypassing (3)	Self-medicating (4)	Self-healing (5)	Difference in Mean		
						(6) = (2)–(3)	(7) = (2)–(4)	(8) = (2)–(5)
***Providers’ characteristics***								
Correct/partially correct medication for the corresponding symptom	0.501 (0.500)	0.509 (0.501)	0.492 (0.502)	0.501 (0.500)	0.486 (0.502)	0.017	0.009	0.024
Full-time formal junior college medical education (yes = 1)	0.063 (0.244)	0.075 (0.265)	0.109 (0.313)	0.052 (0.223)	0.064 (0.246)	−0.034	0.023	0.011
Variety of Western medicine in stock	78.976 (53.826)	96.488 (60.832)	68.180 (48.523)	72.886 (50.366)	83.286 (50.545)	28.308***	23.602***	13.202**
Distance from VC to THC (km)	11.944 (8.209)	12.232 (7.659)	12.328 (8.501)	11.615 (8.328)	13.043 (8.483)	−0.097	0.617	−0.811
Availability of a shuttle from the village to the THC (yes = 1)	0.526 (0.499)	0.528 (0.500)	0.508 (0.502)	0.518 (0.500)	0.593 (0.493)	0.020	0.011	−0.065
Number of clinicians per 1000 people	1.097 (0.793)	1.085 (0.775)	1.164 (0.987)	1.099 (0.809)	1.050 (0.469)	−0.079	−0.014	0.035
Referral rate of patients by the VC	0.088 (0.152)	0.090 (0.156)	0.077 (0.139)	0.092 (0.158)	0.065 (0.107)	0.012	−0.00	0.025*
Number of visits to the VC	395.920 (579.077)	443.671 (569.864)	326.766 (452.189)	381.603 (588.420)	428.636 (635.413)	116.906**	62.07*	15.035

Note: Significance level: *** *p* < 0.01, ** *p* < 0.05, * *p* < 0.1

### Association between quality-related indicators and patients’ healthcare-seeking behaviors

Table 4 presents the results of the multinomial logit regression; the estimators reported are odd ratios. In the case of people seeking medical help, as shown in columns (1) and (3), the clinician’s correct or partially correct medication, (i.e., the unobservable quality indicator) did not influence a patient’s visit to a VC. In contrast, whether the village clinician had received full-time formal junior college medical education((i.e., the observable quality indicator) was positively correlated with the probability of patients visiting a VC. The coefficient was significant at the 5% significance level at 0.441 (column (4)), implying that the probability of visiting a VC compared to self-medicating increased 2.267 times when the village clinician was well-educated. In addition, the observable signal indicator also significantly affected patients’ decisions. The odd ratio of *variety of Western medicine in stock* was statistically significant at 0.420 and 0.627 in columns (1) and (3), respectively. This result implied that the probability of patients’ bypassing and self-medicating compared to the probability of visiting a VC decreased by 58.0 and 37.3%, respectively when the variety of Western medicine in a VC was doubled. However, according to column (5), the odds ratios of the three indicator types were insignificant, which indicated that self-healing patients were insensitive to provider-side factors.

**Table 4:** Determinants of healthcare-seeking behaviors. Base group: seeking healthcare at VCs

	(1)	(2)	(3)	(4)	(5)	(6)
	Bypassing		Self-medicating		Self-healing	
***Providers’ characteristics***						
Correct/partially correct medication	0.891	0.901	1.243	1.320	0.971	1.053
	(0.249)	(0.248)	(0.231)	(0.247)	(0.268)	(0.301)
Senior high school or higher education ×correct/partially correct medication		0.725		0.359*		0.326*
		(0.735)		(0.214)		(0.216)
Whether the clinician has received full-time formal junior college medical education (yes = 1)	0.828	0.826	0.441**	0.438**	0.465	0.459
	(0.362)	(0.360)	(0.172)	(0.172)	(0.307)	(0.305)
Varieties of Western medicine in stock (log)	0.420***	0.419***	0.627***	0.622***	0.676	0.667
	(0.111)	(0.111)	(0.108)	(0.107)	(0.182)	(0.178)
Distance from the VC to the THC (km) (log)	0.983	0.981	0.880	0.874	1.547**	1.532**
	(0.183)	(0.182)	(0.105)	(0.104)	(0.285)	(0.282)
Availability of a shuttle from the village to the THC (yes = 1)	1.194	1.197	1.230	1.236	1.362	1.366
	(0.337)	(0.338)	(0.243)	(0.244)	(0.411)	(0.413)
Number of clinicians per 1000 people	1.278	1.282	1.028	1.035	0.808	0.815
	(0.206)	(0.208)	(0.115)	(0.117)	(0.146)	(0.147)
Referral rate of patients by the VC	0.380	0.377	0.328	0.321	0.228	0.225
	(0.507)	(0.503)	(0.292)	(0.286)	(0.324)	(0.321)
Number of visits to the VC (log)	0.914	0.915	0.893	0.892	0.975	0.975
	(0.111)	(0.110)	(0.0901)	(0.0900)	(0.129)	(0.130)
***Patients’ characteristics***						
Elderly (over 60 = 1)	1.277	1.276	1.131	1.134	1.275	1.277
	(0.362)	(0.361)	(0.218)	(0.218)	(0.377)	(0.379)
Male (yes = 1)	0.840	0.842	1.031	1.047	1.299	1.326
	(0.170)	(0.175)	(0.132)	(0.135)	(0.247)	(0.253)
Senior high school or higher education (yes = 1)	1.232	1.536	1.347	2.349*	2.411**	4.385***
	(0.627)	(1.099)	(0.422)	(1.098)	(1.022)	(2.471)
Hypertension or diabetes (yes = 1)	0.622	0.624	0.544***	0.546***	0.970	0.976
	(0.196)	(0.196)	(0.114)	(0.114)	(0.296)	(0.298)
Non-farm jobholders (yes = 1)	1.310	1.307	0.952	0.945	1.091	1.082
	(0.317)	(0.316)	(0.157)	(0.156)	(0.287)	(0.285)
Village leaders (yes = 1)	0.644	0.653	0.652	0.663	0.856	0.870
	(0.269)	(0.273)	(0.190)	(0.194)	(0.333)	(0.339)
vRelatives of village clinicians (yes = 1)	1.036	1.033	1.137	1.117	0.706	0.690
	(0.441)	(0.440)	(0.336)	(0.330)	(0.326)	(0.320)
Impoverished household (yes = 1)	0.601	0.601	0.600**	0.603**	1.030	1.039
	(0.193)	(0.192)	(0.133)	(0.133)	(0.342)	(0.346)
Moderate symptoms (yes = 1)	1.206	1.205	0.732	0.734	0.181***	0.181***
	(0.417)	(0.417)	(0.147)	(0.147)	(0.0692)	(0.0695)
Severe symptoms (yes = 1)	2.932***	2.938***	0.228***	0.226***	0.0963***	0.0952***
	(0.864)	(0.864)	(0.0443)	(0.0443)	(0.0358)	(0.0356)
Disease fixed effect	YES	YES	YES	YES	YES	YES
County fixed effect	YES	YES	YES	YES	YES	YES
Observations	1578	1578	1578	1578	1578	1578

Notes: a. Significance level: *** *p* < 0.01, ** *p* < 0.05, * *p* < 0.1. Robust standard errors in parentheses. Standard errors are clustered at the household level

b. Owing to some zero values *in the number of visits to the VC,* we used log (1+ initial value) for this variable

We also considered the heterogeneity in patients’ healthcare-seeking behaviors by their highest education level. In the regression model, we added the interaction term between if a household had attained senior high school or higher education and whether the clinician provided correct or partially correct medication in the vignettes. Columns (2), (4), and (6) present the results. The odds ratio of the interaction term *Senior high school or higher education × correct/partially correct medication* was less than one in all three columns and it statistically significant in the last two columns. This result implied that well-educated patients were more likely to be able to identify a clinician’s actual competence and selected a high-quality provider.

### Other factors associated with patients’ healthcare-seeking behaviors

From the patient’s perspective, the severity of their symptom played a crucial role in their health-seeking decision. The probability of bypassing VCs compared to the probability of visiting a VC increased by 193.2% when a patient had a severe symptom (Table 4, column (1)). The relative probability of self-healing compared to the probability of visiting a VC decreased by 90.4% when a patient had a serious symptom (Table 4, column (5)).

Additionally, inadequate economic and social resources forced patients to use village-level PHC. For example, according to Table 4, column (3), patients with hypertension or diabetes preferred to visit a VC instead of self-medicating. Specifically, the probability of chronic patients’ self-medicating compared to the probability of them visiting a VC was 45.6% lower than the general population. Patients from impoverished households also prioritized seeking care at VCs—the probability of those self-medicating compared to those visiting a VC was 40.0% lower than others.

## Discussion

Previous studies on patients’ healthcare-seeking behavior have usually focused on one or two behavior-seeking type, whereas we focused on the following diverse patient behaviors: seeking healthcare at a VC (baseline), bypassing, self-medicating, and self-healing. These behaviors cover all possible patient options to provide a clearer picture of this topic. Moreover, we emphasized VCs and two common symptoms, diarrhea and a cough/ runny nose, wherein patients are supposed to use VCs as their first point of contact. Although VCs provide basic universal PHC for rural inhabitants, most of previous studies have focused on health facilities in urban areas or higher-level facilities in rural areas. Hence, VCs require an exhaustive examination.

Our sample identified the low utilization of village-level PHC. Nearly 24% of the sample regarded VCs as the first point of contact, while approximately 8% were prone to bypassing VCs for better healthcare at higher-level facilities. About 60% chose to self-medicate first, and nearly 9% chose to self-heal. However, it must be noted that diarrhea and a cough/ runny nose symptom are both relatively common and less severe. Therefore, most of the sample was not accustomed to seeking professional healthcare after the initial onset of these symptoms.

Self-medication might worsen antibiotic abuse, which has been considered as the leading cause of antimicrobial resistance (AMR) [40]. AMR poses a global health issue and contributes to substantial health and economic loss [41]. According to the survey in this study, only 33.5% (183/546) of the antibiotics stored in households were prescribed by clinicians, and 60.8% (332/546) were purchased from pharmacies without a prescription. Hence, improvement in village-level PHC also contributes to tackling the inappropriate use of antibiotics in China.

We analyzed the village-level PHC based on the credence nature of healthcare. The healthcare market is part of the credence goods market with information asymmetry between patients and providers. Hence, most patients fail to accurately assess the true quality of providers and, in turn, fail to significantly influence their healthcare-seeking behaviors. We used vignettes as a more advanced and accurate technique to identify the unobservable quality of PHC providers. The findings suggested that rural residents were insensitive to the actual quality of providers because they might have no ability to observe it. This finding is consistent with E Fe, T Powell-Jackson and W Yip [35], who used a 35-item knowledge test to assess the competence of clinicians. Moreover, patients could easily use the observable signal indicator to evaluate the quality of providers, which could subsequently influence their healthcare selection. We controlled a vector of clinic-level characteristics to alleviate the threat of endogenous quality-related indicators on the results. Additionally, we used the two-stage least squares method by instrumenting the measurement of the VC using the average measurement of other VCs within the county. The results were essentially consistent (see Additional file 1, Table A2).

In addition, the data showed that the observable signal indicator was insignificantly associated with the quality indicators (see Additional file 1, Table A3), which implied that there was a significant inconsistency between providers’ competence and patients’ perceptions the quality of care. Moreover, there was no significant correlation between the observable and the unobservable quality indicators. One possible explanation could be the poor quality of providers’ medical education. However, given that much time had passed since most of the clinicians in the sample had completed their education, this might more likely be a result of unbalanced learning efforts during service.

In the credence goods market, information asymmetry varies per person due to differences in individual characteristics. In this regard, we considered the patients’ health literacy, which was defined as the degree to which people obtain, process, and understand the elementary medical information [42] and substantially influenced patients’ healthcare-seeking behaviors. Studies have shown that an individual’s education level significantly influences health literacy both theoretically and empirically [42, 43]. Thus, we used education level as a proxy of patients’ information status, and concluded that the effect of the quality of providers on patients’ healthcare choice was heterogeneous. Patients with a higher education level could more easily identify high-quality PHC than less-educated patient. Further studies should employ more accurate measurements of health literacy to gain concrete empirical evidence in this area.

Our study contributed to the improvement of rural health in China. Specifically, it highlighted that the information asymmetry between patients and providers and the inconsistency between objective healthcare quality and subjective evaluation of patients could impair the effectiveness and efficiency of relevant policies. Therefore, apart from clinician’s formal medical education and in-service training, reforms on village-level PHC should focus on the factors matching patients’ perception of healthcare quality, such as medication availability. In addition, more information disclosure on the quality of PHC is also required to bridge the information gap between patients and providers.

Perceived illness severity significantly affected patients’ healthcare-seeking behaviors. The more severe the symptom, the higher the patients’ tendency to seek formal and professional medical care. Patients with a high socioeconomic status were more inclined to bypass basic-level facilities. These results are consistent with the literature [26]. That low-income patients prefer VCs can be attributed to its affordable pricing [44]. Chronic patients prefer village-level PHC because of their familiarity with the village clinicians. These findings support the indispensability of village-level PHC for vulnerable groups. The current underutilization of VCs poses a threat to the supply of village-level PHC in the future because low demand might lead to a “healthcare desert” [7]. People with low socioeconomic status are risk-takers, therefore, inequality within rural areas may increase over time, which highlights the need to address and resolve the current marginalization of VCs.

This study had potential limitations owing to the study design and sample selection. First, although the survey asked patients about their last experience of healthcare-seeking, the symptom recall period was one year, which is much longer than the period usually used in the literature (i.e., two weeks or one month). Thus, potential recall bias from the long recall period might have caused inaccuracy in the estimates. Second, Table A1 shows, differences between the included and excluded samples, which might bias the estimates of the factors influencing healthcare-seeking behaviors. Finally, the sample was limited to 3 prefectures in Yunnan province. Therefore, it is essential to conduct multi-centrality research encompassing diverse regions and nationalities in the future.

## Conclusions

A functioning PHC system is crucial for both the whole national health system and population health outcomes. However, many patients, not only in LMICs but in high-income countries, prefer to seek care at high-level facilities or self-medicate rather than visit a PHC provider, causing the underutilization of PHC and inefficiency of the PHC system. Healthcare quality is a major concern for patients’ healthcare-seeking choices. Therefore, this study explored the factors associated with healthcare-seeking behaviors, especially the unobservable quality indicator, the observable quality indicator, and the observable signal indicator. We focused on diarrhea and a cough/ runny nose, which can be diagnosed and treated in VCs, the bottom-level PHC providers in rural China. We highlighted the credence attribute of PHC and the inherent information asymmetry between patients and providers. In this regard, we clarified that patients could not precisely identify the actual quality of PHC measured by clinical vignettes, while they were more likely to make decisions based on the observable signal indicator, the availability of medical resources. This inconsistency between the objective quality of providers and the quality perceived by patients will weaken the effectiveness and efficiency of reforms aimed at improving the quality and utilization of PHC and enhancing the health outcomes of rural populations. Therefore, the quality of PHC should be more observable to patients. Establishing an authoritative PHC quality evaluation system based on government credibility may be an effective solution. Our results also indicated that people from low socioeconomic backgrounds were more likely to visit PHC providers, which further highlights the importance of PHC for promoting the welfare of the vulnerable.

## Supplementary Information

**Additional file 1.**

## Data Availability

The datasets used and/or analyzed during the current study are available from the corresponding author on reasonable request.

## Abbreviations

PHC: Primary health care
LMICs: Low- and middle-income countries
VCs: Village clinics
CHs: County hospitals
THCs: Township health centers.

## Footnotes

1 We selected eight households on the first day of the follow-up survey. However, unexpected bad weather conditions limited our transportation inside the village. Since this hurdle would have made it difficult for the enumerators to visit eight households, only six households were surveyed each day on the second day of the follow-up survey.

2 Two of the villages included seven households, while one village included four households.
