# Supplementary material for: Measurements of quality of village-level care and patients’ healthcare-seeking behaviors in rural China
Source: BMC Public Health. 2021 Oct 17;21:1873. doi: 10.1186/s12889-021-11946-8 (PMC8520638; doi:10.1186/s12889-021-11946-8)
Supplement: Supplementary file 1 — Additional file 1. [file 12889_2021_11946_MOESM1_ESM.docx]

**Table A1**. Comparison of providers and patients’ characteristics in the Included and Excluded Episodes *(mean)*

|  | Included  (1) | Excluded  (2) | Difference  (3)=(1)-(2) |
| --- | --- | --- | --- |
| N (%) | 1578  (88.5%) | 205  (11.5%) |  |
| ***Patients characteristics*** |  |  |  |
| Elderly (over 60) | 0.277 | 0.341 | -0.065* |
| Male (yes=1) | 0.497 | 0.449 | 0.049 |
| Education |  |  |  |
| Illiterate | 0.302 | 0.322 | -0.020 |
| Primary school | 0.369 | 0.405 | -0.036 |
| Junior middle school | 0.256 | 0.180 | 0.076** |
| Senior middle school | 0.048 | 0.068 | -0.021 |
| College | 0.026 | 0.024 | 0.002 |
| Hypertension or diabetes (yes=1) | 0.163 | 0.249 | -0.086*** |
| Non-farm jobholder (yes=1) | 0.435 | 0.478 | -0.043 |
| Village leader (yes=1) | 0.073 | 0.088 | -0.015 |
| Self-reported severity of a symptom |  |  |  |
| Mild | 0.511 | 0.229 | 0.282*** |
| Moderate | 0.234 | 0.234 | -0.000 |
| Severe | 0.255 | 0.537 | -0.281*** |
| Relative of village clinicians (yes=1) | 0.132 | 0.180 | -0.049* |
| Impoverished household (yes=1) | 0.313 | 0.298 | 0.015 |
| ***Provider characteristics*** |  |  |  |
| Correct/partially correct medication for the corresponding symptom | 0.501 | 0.498 | 0.003 |
| Whether the clinician has received full-time formal junior college medical education (yes=1) | 0.063 | 0.029 | 0.034* |
| Variety of Western medicine at stock | 78.976 | 79.205 | -0.229 |
| Distance from the VC to the THC (km) | 11.944 | 11.802 | 0.142 |
| Availability of a shuttle from village to THC (yes=1) | 0.526 | 0.483 | 0.043 |
| Number of clinicians per 1000 people | 1.097 | 1.060 | 0.037 |
| Referral rate of patients by the VC | 0.088 | 0.056 | 0.031*** |
| Number of visits to the VC | 395.920 | 485.029 | -89.109** |

Note: Significance level: *** p<0.01, ** p<0.05, * p<0.1.

**Table A2**. Correlation between quality-related indicators and healthcare-seeking behaviors. Base group: seeking healthcare at VCs (IV/2SLS method)

|  | (1) | (2) | (3) |
| --- | --- | --- | --- |
|  | Bypassing | Self-medicating | Self-healing |
| ***Provider characteristics*** |  |  |  |
| Correct/partially correct medication  for the corresponding symptom | 0.016 | -0.008 | -0.052 |
|  | (0.074) | (0.054) | (0.080) |
| Whether the clinician has received full-time formal junior college medical education (yes=1) | -0.053 | -0.160 | -0.242* |
|  | (0.115) | (0.101) | (0.146) |
| Varieties of Western medicines in stock (log) | -0.145*** | -0.063* | -0.132** |
|  | (0.055) | (0.035) | (0.060) |
| Provider controls | YES | YES | YES |
| Patient controls | YES | YES | YES |
| Disease fixed effect | YES | YES | YES |
| County fixed effect | YES | YES | YES |
|  |  |  |  |
| Observations | 493 | 1,288 | 506 |

Note: Significance level: *** p<0.01, ** p<0.05, * p<0.1.

**Table A3.** Correlation between the observable signal indicator and the quality indicators

|  | (1) | (2) | (3) |
| --- | --- | --- | --- |
|  | Correct/partially correct medication for the corresponding symptom | Whether the clinician has received full-time formal junior college medical education (yes=1) | Correct/partially correct medication for the corresponding symptom |
| Variety of Western medicine in stock (log) | 0.059 | -0.040 |  |
|  | (0.087) | (0.038) |  |
| Whether the clinician has received a full-time formal junior college medical education (yes=1) |  |  | -0.017 |
|  |  |  | (0.195) |
|  |  |  |  |
| Provider controls | YES | YES | YES |
| County fixed effect | YES | YES | YES |
| Observations | 114 | 114 | 114 |

Note：Significance level: *** p<0.01, ** p<0.05, * p<0.1.
